# Expansion of the multidrug-resistant clonal complex 320 among invasive *Streptococcus pneumoniae* serotype 19A after the introduction of a ten-valent pneumococcal conjugate vaccine in Brazil

**DOI:** 10.1371/journal.pone.0208211

**Published:** 2018-11-29

**Authors:** Ana Paula Cassiolato, Samanta Cristine Grassi Almeida, Ana Lúcia Andrade, Ruth Minamisava, Maria Cristina de Cunto Brandileone

**Affiliations:** 1 National Laboratory for Meningitis and Pneumococcal Infections, Center of Bacteriology, Adolfo Lutz Institute, São Paulo, State of São Paulo, Brazil; 2 Institute of Tropical Pathology and Public Health, Federal University of Goiás, Goiania, State of Goiás, Brazil; 3 Faculty of Nursing, Federal University of Goiás, Goiânia, State of Goiás, Brazil; Carnegie Mellon University, UNITED STATES

## Abstract

**Background:**

In 2010, a ten–valent pneumococcal conjugate vaccine (PCV10) was introduced in the routine infant national immunization program in Brazil. Invasive pneumococcal disease (IPD) caused by serotype 19A (Spn19A) increased after the introduction of PCVs in several countries. We compared the frequency, antimicrobial resistance and molecular patterns of invasive Spn19A strains before and after PCV10 introduction in Brazil using data from the national laboratory-based surveillance.

**Methods:**

We analyzed invasive Spn19A strains isolated from 2005–2009 (pre-PCV10 period), 2011–2015 and 2016–2017 (post-PCV10 periods). Antimicrobial susceptibility was performed for all Spn19A strains, and multilocus sequence typing (MLST) was performed for strains isolated in the age groups <5 years and ≥50 years.

**Results:**

Among the study period, a total of 9,852 invasive Spn strains were analyzed, and 673 (6.8%) belonged to serotype 19A. Overall, the proportion of Spn19A among the total number of IPD strains increased from 2.8% in 2005–2009 to 7.0% and 16.4% in 2011–2015 and 2016–2017, respectively. The relative increase in Spn19A was observed especially in children <5 years old (2005–2009: 3.2%; 2011–2015: 15.5%; 2016–2017: 31.2%). The percentage of penicillin resistance (MIC 2.0–4.0 μg/mL), erythromycin resistance and multidrug resistance (MDR) increased after PCV10 introduction due to the expansion of the MDR clonal complex CC320 (2005–2009: 8.6%; 2011–2015: 56.1%; 2016–2017: 66.5%).

**Conclusion:**

We observed an expansion of MDR-CC320 among invasive Spn19A strains after PCV10 introduction in Brazil, probably related to a combination of factors, such as vaccination and antimicrobial pressure. Continued surveillance of Spn19A strains is necessary to monitor the sustainability of this clonal complex in the Brazilian population.

## Introduction

*Streptococcus pneumoniae* (Spn) is an important cause of invasive infectious diseases, especially in young children and the elderly [1,2]. Although the widespread use of pneumococcal conjugate vaccines (PCV) has reduced the incidence of invasive pneumococcal disease (IPD) caused by vaccine types, the emergence of nonvaccine types has been a concern [3–6]. Serotype 19A (Spn19A) became the most common cause of IPD in several countries after the introduction of the 7-valent pneumococcal conjugate vaccine (PCV7) and, importantly, it has been the most common serotype related to antimicrobial resistance [7–12].

In the USA, the incidence of IPD caused by Spn19A in children <5 years old increased from 2.6 to 11.1 cases/100,000 after 7 years of PCV7 use [13], but the incidence decreased after the introduction of PCV13, which targets this serotype [14]. An increase in the prevalence of IPD caused by Spn19A also occurred in countries that introduced the 10-valent pneumococcal conjugate vaccine (PCV10) [15]. In Finland, where PCV10 was introduced in 2010, Spn19A represented over half of the total IPD cases in children <5 years old in 2015 [16,17]. In Chile, Spn19A was identified in 4–8% of IPD cases in children <2 years old before PCV10, and it increased to 19–25% after 3–4 years of PCV10 use [18].

In contrast, an increase in Spn19A as a cause of IPD in South Korea and as a cause of acute otitis media in Israel was reported before PCV7 introduction in these countries [19,20]. In the United Kingdom, a rapid decline of IPD caused by serotype 19A was observed after PCV13 introduction; however, a modest increase in serotype 19A, especially among adults over 65 years, has been recently observed, but the number of serotype 19A cases still remains below the pre-PCV13 levels [21]. Therefore, studies on serotype 19A after vaccination are still controversial, and it is an important issue to investigate over a longer period of time.

Studies on the molecular characterization of Spn19A strains from many settings have demonstrated that five major clonal complexes (CC) are related to serotype 19A, specifically CC81, CC193, CC199, CC276 and CC320 [22]. Increased antimicrobial and multidrug resistance in Spn19A were mainly related to the emergence of CC320 after vaccination [7, 23–25].

In March 2010, PCV10 was introduced in the routine infant national immunization program in Brazil, using a 3+1 dose schedule (at 2, 4 and 6 months of age plus a booster at 12–18 months); a catch-up with the first two doses plus a booster was also offered for children aged 7–11 months, and a single dose was offered for children aged 12–23 months [26]. In 2016, the PCV10 schedule changed to 2+1 doses (at 2 and 4 months plus a booster at 12 months) [27].

The characterization of Spn19A from Brazil has been described only in independent studies, each with a limited number of Spn19A strains isolated [28–30]. In a recent report, using data from Brazilian laboratory-based surveillance, nonvaccine types increased, with Spn19A being the most prevalent cause of IPD after four years of PCV10 use [31]. To date, there is a lack of comprehensive long-term analysis of Spn19A at a national level, which led us to investigate the frequency over a longer period of time (seven years after PCV10) and the characteristics of the Spn19A strains isolated in Brazil.

We herein present the frequency, antimicrobial resistance and molecular patterns of invasive *S*. *pneumoniae* serotype 19A strains before and after PCV10 introduction in Brazil, using data from national laboratory-based surveillance from 2005 to 2017.

## Material and methods

### Collection of strains

Pneumococcal strains are routinely sent to the Center of Bacteriology at the Adolfo Lutz Institute (IAL), which is the national reference laboratory for bacterial meningitis and IPD in Brazil. IAL receives Spn strains collected from 25 public health laboratories located in 25 Brazilian states, composing the national laboratory-based passive surveillance system, coordinated by the Ministry of Health. Spn19A strains isolated from January 2005 to December 2009 and January 2011 to December 2017 were included in the study. The year 2010 was excluded from the study, because it was the year of PCV10 introduction. The Ethics Committee of the Adolfo Lutz Institute approved the study.

### Microbiology methods

At IAL, pneumococcal strains were confirmed by standard methods [32]. Strains were serotyped by the Pneumotest-latex agglutination kit and by the Quellung reaction with antisera from the Statens Serum Institute (Copenhagen, Denmark) [33]. Penicillin (PEN) susceptibility was performed by screening for susceptibility to oxacillin (OXA) using disk diffusion. The disk diffusion method was also performed for chloramphenicol (CHL), erythromycin (ERY), trimethoprim-sulphamethoxazole (STX) and vancomycin (VAN) according to the Clinical Laboratory Standards Institute guidelines (CLSI, 2016) [34].

Strains with OXA resistance (halo ≤19 mm) were analyzed for minimum inhibitory concentration (MIC) by broth microdilution to PEN [34]. PEN nonmeningeal oral breakpoints were used to classify the strains as susceptible (MIC ≤0.06 μg/ml), intermediate (MIC 0.12–1.0 μg/ml) or resistant (MIC 2.0–4.0 μg/ml) [34]. These breakpoints usually reflect PBP gene alterations that confer PEN MICs above the basal typical value of pneumococcal susceptible strains [35,36]. Multidrug resistance (MDR) was defined as nonsusceptibility (intermediate or resistant) to at least three antibiotic classes [36] from among the five antibiotics tested (PEN, CHL, ERY, STX and VAN).

### Molecular characterization

Multilocus sequence typing (MLST) was performed according to Enright and Spratt [37]. The allele number and sequence type (ST) were assigned using the pneumococcal MLST webpage [38]; new alleles and profiles were submitted to the MLST website curator for allele number and ST assignment [38]. Clonal complexes (CC) were defined using eBURST software [39], where the STs that shared five of the seven alleles with at least one other ST in the complex were included in the same CC. The CC analysis was performed using the STs found in this study.

### Data analysis

Only one invasive Spn strain from each patient was included in the analysis. Different serotypes isolated >30 days apart from the same patient were considered different IPD episodes. Invasive Spn19A frequency and antimicrobial resistance were investigated for three age groups (children under 5 years old, individuals 5–49 years old and individuals ≥50 years old) over three periods of time: 2005–2009 (pre-PCV10), 2011–2015 and 2016–2017 (post-PCV10 periods). For molecular characterization, we studied only the strains isolated from the two age groups with the highest incidence of IPD: <5 years and ≥50 years.

For a more comprehensive comparative analysis of frequency, antimicrobial susceptibility and molecular characterization of the serotype 19A strains in the same age groups and periods we used recently published data for Spn19A frequency from 2005–2009 (pre-PCV10) and 2011–2015 (post-PCV10) [31]. However, we used different age groupings (<5 years, 5–49 years and ≥50 years) and excluding the year 2010 from this study. The same inclusion/exclusion criteria for strains were used in both studies.

The frequency of Spn19A was calculated as a percentage by the number of invasive serotype 19A strains divided by the total number of invasive Spn strains. The percentage change was used to indicate the change in the percentage frequency of Spn19A and the change in the percentage of the antimicrobial resistance criteria between 2011–2015 or 2016–2017 (post-PCV10 periods) and 2005–2009 (pre-PCV10 period) using the following formula: [(percentage of Spn19A in the post-PCV10 period–percentage of Spn19A in the pre-PCV10 period / percentage of Spn19A in the pre-PCV10 period) * 100]. A positive result of percentage change expresses a relative increase in the post-PCV10 period, whereas a negative result of percentage change expresses a relative reduction in the postvaccination period.

The Chi-squared test for trends was used to analyze the period time trends regarding invasive Spn19A for each age group. Differences in the proportions of antibiotic resistance of invasive Spn19A between periods of time were identified using Chi-square tests. *P-values* <0.05 were considered statistically significant.

## Results

A total of 9,852 IPD strains, recovered from a normally sterile site, were received at the IAL in the study periods. Among these strains, 6.8% (673/9,852) belonged to serotype 19A. A total of 254 (37.7%, 254/673) Spn19A strains were obtained from meningitis cases, with 227 (89.4%, 227/254) strains isolated from cerebrospinal fluid and 27 (10.6%, 27/254) from blood; 419 (62.3%, 419/673) strains were from nonmeningitis cases, with 350 (83.5%, 350/419) strains isolated from blood and 69 (16.5%, 69/419) from other sterile sites.

Table 1 shows the distribution of Spn19A by age group in 2016–2017, plus the distributions previously reported from 2005–2009 and 2011–2015 [31]. The proportion of Spn19A among the total number of IPD strains increased over the periods in all age groups, with the highest increase (875.3%) of Spn19A in children <5 years in 2016–2017 (Table 1).

**Table 1:** Distribution of invasive *Streptococcus pneumoniae* serotype 19A (n = 673) by age group (<5 years, 5–49 years and ≥50 years) in the pre-PCV10 period (2005–2009) and in the post-PCV10 periods (2011–2015 and 2016–2017).

Age group	Spn	2005–2009Ɨ	2011–2015Ɨ		2016–2017		χ^2^ for trend	*p-value*
		no. (%)	no. (%)	% change*	no. (%)	% change*		
<5 years	19A	44 (3.2)	126 (15.5)	384.7	92 (31.2)	875.3	230.0	**<0.001**
	total	1,376	813		295			
5–49 years	19A	43 (2.5)	104 (5.2)	106.6	87 (14.7)	484.1	105.1	**<0.001**
	total	1,712	2,004		593			
≥50 years	19A	17 (2.5)	87 (5.1)	106.3	73 (11.3)	359.9	54.8	**<0.001**
	total	693	1,719		647			
All ages	19A	104 (2.8)	317 (7.0)	154.1	252 (16.4)	496.9	296.2	**<0.001**
	total	3,781	4,536		1,535			

19A: total number of invasive *Streptotoccus pneumoniae* serotype 19A in the period; total: total number of invasive *Streptococcus pneumoniae* strains in the period; bold: p-*value* <0.05 was considered statistically significant;

* Pre-PCV10 period used as reference;

^Ɨ^ Inclusion of frequency of Spn19A previously reported [31].

For antimicrobial susceptibility, 99.7% (671/673) of the Spn19A strains were tested (Table 2). Spn19A showed antimicrobial resistance in 2005–2009 (pre-PCV10 period), but we observed a relative increase of PEN resistant (MIC of 2.0–4.0 μg/ml), ERY resistant and MDR resistant strains after PCV10 vaccination with the highest percentages in children <5 years from 2016–2017 (PEN-R: 71.4%, 65/91; ERY-R: 85.7%, 78/91; MDR: 79.1%, 72/91); a high frequency of resistance to STX was observed in all three study periods (Table 2).

**Table 2:** Distribution of antimicrobial susceptibility of invasive *Streptococcus pneumoniae* serotype 19A (n = 671) by age group (<5 years, 5–49 years and ≥50 years) in the pre-PCV10 period (2005–2009) and in the post-PCV10 periods (2011–2015 and 2016–1017) in Brazil.

Age group	Period	no.	Antibiotic Susceptibility Pattern									
			PEN			ERY		STX			MDR	
			S	I	R	S	R	S	I	R	No	Yes
			no. (%)	no. (%)	no. (%)	no. (%)	no. (%)	no. (%)	no. (%)	no. (%)	no. (%)	no. (%)
<5 years	2005–2009	44	14 (31.8)	25 (56.8)	5 (11.4)	35 (79.5)	9 (20.5)	8 (18.2)	7 (15.9)	29 (65.9)	36 (81.8)	8 (18.2)
	2011–2015	125Ɨ	18 (14.4)	27 (21.6)	80 (64.0)	33 (26.4)	92 (73.6)	8 (6.4)	14 (11.2)	103 (82.4)	37 (29.6)	88 (70.4)
	% change*		*-54*.*7*	*-62*.*0*	*463*.*2*	*-66*.*8*	*259*.*8*	*-64*.*8*	*-29*.*6*	*25*.*0*	*-63*.*8*	*287*.*2*
	*p-value*			*0*.*699*	***<0*.*001***		***<0*.*001***		*0*.*308*	***0*.*014***		***<0*.*001***
	2016–2017	91Ɨ	12 (13.2)	14 (15.4)	65 (71.4)	13 (14.3)	78 (85.7)	6 (6.6)	6 (6.6)	79 (86.8)	19 (20.9)	72 (79.1)
	% change*		*-58*.*6*	*-72*.*9*	*528*.*6*	*-82*.*0*	*319*.*0*	*-63*.*7*	*-58*.*6*	*31*.*7*	*-74*.*5*	*335*.*2*
	*p-value*			*0*.*408*	***<0*.*001***		***<0*.*001***		*0*.*863*	***0*.*020***		***<0*.*001***
5–49 years	2005–2009	43	23 (53.5)	19 (44.2)	1 (2.3)	36 (83.7)	7 (16.3)	8 (18.6)	8 (18.6)	27 (62.8)	39 (90.7)	4 (9.3)
	2011–2015	104	20 (19.2)	30 (28.8)	54 (51.9)	46 (44.2)	58 (55.8)	21 (20.2)	9 (8.7)	74 (71.2)	54 (51.9)	50 (48.1)
	% change*		*-64*.*0*	*-34*.*7*	*2*,*132*.*7*	*-47*.*2*	*242*.*6*	*8*.*5*	*-53*.*5*	*13*.*3*	*-42*.*8*	*416*.*8*
	*p-value*			*0*.*157*	***<0*.*001***		***<0*.*001***		*0*.*181*	*0*.*927*		***<0*.*001***
	2016–2017	87	15 (17.2)	22 (25.3)	50 (57.5)	26 (29.9)	61 (70.1)	13 (14.9)	9 (10.3)	65 (74.7)	32 (36.8)	55 (63.2
	% change*		*-67*.*8*	*-42*.*8*	*2*,*371*.*3*	*-64*.*3*	*330*.*7*	*-19*.*7*	*-44*.*4*	*19*.*0*	*-59*.*4*	*579*.*6*
	*p-value*			*0*.*207*	***<0*.*001***		***<0*.*001***		*0*.*578*	*0*.*434*		***<0*.*001***
≥50 years	2005–2009	17	7 (41.2)	9 (52.9)	1 (5.9)	13 (76.5)	4 (23.5)	3 (17.6)	5 (29.4)	9 (52.9)	13 (76.5)	4 (23.5)
	2011–2015	87	10 (11.5)	28 (32.2)	49 (56.3)	29 (33.3)	58 (66.7)	14 (16.1)	10 (11.5)	63 (72.4)	35 (40.2)	52 (59.8)
	% change*		*-72*.*1*	*-39*.*2*	*857*.*5*	*-56*.*4*	*183*.*3*	*-8*.*8*	*-60*.*9*	*36*.*8*	*-47*.*4*	*154*.*0*
	*p-value*			*0*.*208*	***<0*.*001***		***<0*.*001***		*0*.*308*	*0*.*576*		***0*.*006***
	2016–2017	73	8 (11.0)	22 (30.1)	43 (58.9)	17 (23.3)	56 (76.7)	12 (16.4)	4 (5.5)	57 (78.1)	25 (34.2)	48 (65.8)
	% change*		*-73*.*4*	*-43*.*1*	*901*.*4*	*-69*.*5*	*226*.*0*	*-6*.*8*	*-81*.*4*	*47*.*5*	*-55*.*2*	*179*.*5*
	*p-value*			*0*.*240*	***<0*.*001***		***<0*.*001***		*0*.*074*	*0*.*531*		***0*.*002***
All ages	2005–2009	104	44 (42.3)	53 (51.0)	7 (6.7)	84 (80.8)	20 (19.2)	19 (18.3)	20 (19.2)	65 (62.5)	88 (84.6)	16 (15.4)
	2011–2015	316Ɨ	48 (15.2)	85 (26.9)	183 (57.9)	108 (34.2)	208 (65.8)	43 (13.6)	33 (10.4)	240 (75.9)	126 (39.9)	190 (60.1)
	% change*		*-64*.*1*	*-47*.*2*	*760*.*4*	*-57*.*7*	*242*.*3*	*-25*.*5*	*-45*.*7*	*21*.*5*	*-52*.*9*	*290*.*8*
	*p-value*			*0*.*157*	***<0*.*001***		***<0*.*001***		*0*.*423*	*0*.*111*		***<0*.*001***
	2016–2017	251Ɨ	35 (13.9)	58 (23.1)	158 (62.9)	56 (22.3)	195 (77.7)	31 (12.4)	19 (7.6)	201 (80.1)	76 (30.3)	175 (69.7)
	% change*		*-67*.*0*	*-54*.*7*	*835*.*2*	*-72*.*4*	*304*.*0*	*-32*.*4*	*-60*.*6*	*28*.*1*	*-64*.*2*	*353*.*2*
	*p-value*			*0*.*281*	***<0*.*001***		**<*0*.*001***		*0*.*211*	***0*.*046***		***<0*.*001***

PEN S: penicillin susceptible (MIC ≤0.06 μg/mL); PEN I: penicillin intermediate (MIC 0.12–1.0 μg/mL); PEN R: penicillin resistant (MIC 2.0–4.0 μg/mL); ERY: erythromycin; STX: trimethoprim-sulphamethoxazole; S: susceptible; I: intermediate; R: resistant; MDR: multidrug-resistant; bold: *p value* <0.05 was considered statistically significant;

* Pre-PCV10 period used as reference;

^Ɨ^ One strain was not viable for analyzes.

Only two Spn19A strains showed resistance to CHL (one isolated in children <5 years in 2007 and one isolated in the age group 5–49 years in 2014) and none to VAN. MDR strains were identified for the first time in 2007, the pre-PCV10 period, with 99.5% (379/381) of the strains expressing a pattern of nonsusceptibility to PEN, ERY and STX. From 2016–2017, MDR-Spn19A represented 80.3% (175/218) of all MDR in invasive *S*. *pneumoniae* (S1 Table).

From all the Spn19A strains (n = 673), 439 were isolated from <5 years (n = 262) and ≥50 years (n = 177). MLST was performed for 399 Spn19A strains from these two age groups (<5 years: n = 243; ≥50 years: n = 156; 40 strains were not viable). Table 3 shows the frequency of CC/ST and the related antimicrobial susceptibility pattern of invasive Spn19A by age groups and study periods. Overall, 60 STs (25 STs first detected in this study) and 7 CCs were identified. Four main CCs comprised 86.2% (344/399) of the Spn19A strains (CC320: n = 213; CC1118: n = 63; CC276: n = 39; CC733: n = 29).

**Table 3:** Distribution of clonal complex, sequence type and antimicrobial susceptibility pattern of invasive *Streptococcus pneumoniae* serotype 19A (n = 399) by age group (<5 years and ≥50 years) in the pre-PCV10 period (2005–2009) and in the post-PCV10 periods (2011–2015 and 2016–2017) in Brazil.

CC	2005–2009					2011–2015					2016–2017				
	<5 years	≥50 years	total	Antimicrobial Susceptibility Pattern		<5 years	≥50 years	total	Antimicrobial Susceptibility Pattern		<5 years	≥50 years	total	Antimicrobial Susceptibility Pattern	
ST				S	I + R				S	I+R				S	I+R
	no. (%)	no. (%)	no. (%)	no.	(no.)	no. (%)	no. (%)	no. (%)	no.	(no.)	no. (%)	no. (%)	no. (%)	no.	(no.)
CC320	5 (11.6)	0 (0.0)	5 (8.6)	0	**PEN-ERY-STX (4)**; ERY-STX (1)	64 (58.2)	37 (52.8)	101 (56.1)	0	**PEN-ERY-STX (100)**; PEN-STX (1)	64 (71.1)	43 (60.6)	107 (66.5)	0	**PEN-ERY-STX (105)**; ERY-STX (2)
320	4 (9.3)	0 (0.0)	4 (6.9)	0	**PEN-ERY-STX (4)**	58 (52.7)	32 (45.7)	90 (50.0)	0	**PEN-ERY-STX (89)**; PEN-STX (1)	54 (60.0)	37 (52.1)	91 (56.5)	0	**PEN-ERY-STX (89)**; ERY-STX (2)
1451	0 (0.0)	0 (0.0)	0 (0.0)	0	0	3 (2.7)	0 (0.0)	3 (1.7)	0	**PEN-ERY-STX (3)**	2 (2.2)	0 (0.0)	2 (1.2)	0	**PEN-ERY-STX (2)**
other 15†	1 (2.3)	0 (0.0)	1 (1.7)	0	ERY-STX (1)	3 (2.7)	5 (7.1)	8 (4.4)	0	**PEN-ERY-STX (8)**	8 (8.9)	6 (8.4)	14 (8.7)	0	**PEN-ERY-STX (14)**
CC1118	23 (53.5)	9 (60.0)	32 (55.2)	2	PEN-STX (24); STX (5); PEN (1)	13 (11.8)	9 (12.9)	22 (12.2)	0	**PEN-ERY-STX (1);** PEN-STX (17); STX (2); PEN (2)	3 (3.3)	6 (8.4)	9 (5.6)	0	PEN-STX (5); PEN (2); STX (2)
1118	9 (20.9)	6 (40.0)	15 (25.9)	2	PEN-STX (10); STX (3)	2 (1.8)	3 (4.3)	5 (2.8)	0	PEN-STX (3); STX (2)	2 (2.2)	2 (2.8)	4 (2.5)	0	PEN-STX (3); STX (1)
2878	7 (16.3)	0 (0.0)	7 (12.1)	0	PEN-STX (6); PEN (1)	2 (1.8)	2 (2.9)	4 (2.2)	0	**PEN-ERY-STX (1)**; PEN-STX (3)	1 (1.1)	2 (2.8)	3 (1.9)	0	PEN-STX (1); PEN (1); STX (1)
2880	4 (9.3)	1 (6.7)	5 (8.6)	0	PEN-STX (3); STX (2)	1 (0.9)	2 (2.9)	3 (1.7)	0	PEN-STX (2); PEN (1)	0 (0.0)	0 (0.0)	0 (0.0)	0	0
9837*	0 (0.0)	0 (0.0)	0 (0.0)	0	0	3 (2.7)	2 (2.9)	5 (2.8)	0	PEN-STX (5)	0 (0.0)	1 (1.4)	1 (0.6)	0	PEN-STX (1)
other 7‡	3 (7.0)	2 (13.3)	5 (8.6)	0	PEN-STX (5)	5 (4.5)	0 (0.0)	5 (2.8)	0	PEN-STX (4); PEN (1)	0 (0.0)	1 (1.4)	1 (0.6)	0	PEN (1)
CC276	3 (7.0)	3 (20.0)	6 (10.3)	0	**PEN-ERY-STX (5)**; **PEN-ERY-CHL (1)**	8 (7.3)	7 (10.0)	15 (8.3)	0	**PEN-ERY-STX (12)**; PEN-ERY (3)	10 (11.1)	8 (11.3)	18 (11.2)	0	**PEN-ERY-STX (10)**; PEN-ERY (8)
276	3 (7.0)	3 (20.0)	6 (10.3)	0	**PEN-ERY-STX (5)**; **PEN-ERY-CHL (1)**	6 (5.4)	6 (8.6)	12 (6.7)	0	**PEN-ERY-STX (10)**; PEN-ERY (2)	7 (7.8)	7 (9.9)	14 (8.7)	0	**PEN-ERY-STX (8)**; PEN-ERY (6)
other 2§	0 (0.0)	0 (0.0)	0 (0.0)	0	0	2 (1.8)	1 (1.4)	3 (1.7)	0	**PEN-ERY-STX (2)**; PEN-ERY (1)	3 (3.3)	1 (1.4)	4 (2.5)	0	**PEN-ERY-STX (2)**; PEN-ERY (2)
CC733	3 (7.0)	1 (6.7)	4 (6.9)	1	STX (3)	10 (9.1)	6 (8.6)	16 (8.9)	4	STX (12)	7 (7.8)	2 (2.8)	9 (5.6)	1	STX (8)
733	2 (4.7)	1 (6.7)	3 (5.2)	1	STX (2)	8 (7.3)	6 (8.6)	14 (7.8)	4	STX (10)	7 (7.8)	2 (2.8)	9 (5.6)	1	STX (8)
other 3¶	1 (2.3)	0 (0.0)	1 (1.7)	0	STX (1)	2 (1.8)	0 (0.0)	2 (1.1)	0	STX (2)	0 (0.0)	0 (0.0)	0 (0.0)	0	0
Other 3 CCs#	8 (18.6)	1 (6.7)	9 (15.5)	2	**PEN-ERY-STX (2)**; STX (4); PEN (1)	11 (10.0)	5 (7.1)	16 (8.9)	2	**PEN-ERY-STX (9)**; PEN-STX (2); STX (2); ERY-STX (1)	5 (5.5)	5 (7.0)	10 (6.2)	2	**PEN-ERY-STX (4)**; PEN-STX (1); STX (1); ERY-STX (1), ERY (1)
Singletons**	1 (2.3)	1 (6.7)	2 (3.4)	0	PEN-STX (1); STX (1)	4 (3.6)	6 (8.6)	10 (5.5)	2	PEN- STX (3); STX (3); PEN-ERY (2);	1 (1.1)	7 (9.9)	8 (5.0)	1	PEN-STX (4); STX (1); PEN-ERY (1); PEN (1)
Total	43	15	58	5	**PEN-ERY-STX (11)**; **PEN-ERY-CHL (1)**; PEN-STX (25); STX (13); PEN (2); ERY-STX (1)	110	70	180	8	**PEN-ERY-STX (122)**; PEN-STX (23); STX (19); PEN-ERY (5); PEN (2); ERY-STX (1)	90	71	161	4	**PEN-ERY-STX (119)**; STX (12); PEN-STX (10); PEN-ERY (9); PEN (3); ERY-STX (3); ERY (1)

CC: clonal complex; ST: sequence type; S: susceptible to all antibiotics tested; I: intermediate; R: resistant; PEN: penicillin; ERY: erythromycin; STX: STX: trimethoprim-sulphamethoxazole; CHL: chloramphenicol; bold: multidrug-resistant strains;

* new STs;

^†^ STs 9796* (n = 4), 11485*, 12078 (n = 3 each), 11936* (n = 2), 202, 237, 271, 4768, 8549, 8884, 11486*, 13290, 13650*, 13651*, 13683* (n = 1 each);

^‡^ STs 9943* (n = 3), 7034, 9838* (n = 2 each), 2260, 9799*, 9941*, 9942* (n = 1 each);

^§^ STs 2674 (n = 4), 11935* (n = 3);

^¶^ STs 9797*, 9839*, 11331* (n = 1 each);

^#^ CC9793 [n = 21: STs 8640 (n = 9), 9793* (n = 5), 9801 (n = 3), 11933* (n = 2), 9794*, 9795* (n = 1 each)], CC199 [n = 12: STs 876 (n = 4), 199 (n = 3), 416 (n = 2), 667, 1756, 11484* (n = 1 each)], CC387-1131 [n = 2: STs 387, 1131 (n = 1 each)];

** 63, 2013, 9836* (n = 3 each); 66, 156, 4913 (n = 2 each), 796, 2345, 9940*, 11327, 11934* (n = 1 each).

CC320 was the most frequent CC in this study, represented by 53.4% (213/399) of the Spn19A strains. From 2005–2009 (pre-PCV10), 5 strains (11.6%, 5/43) belonging to CC320 were identified in the <5 years age group. However, since PCV10 use, CC320 became the most common CC among Spn19A strains in both age groups and time periods. CC320 had a great diversity of ST (n = 17). ST320 was the most representative ST (86.9%, 185/213) in this CC and was responsible for the expansion of CC320 after PCV10 vaccination (Table 3).

CC320 was related to MDR starting in the pre-PCV10 period, showing higher rates after PCV10 introduction [2011–2015: 99.0% (100/101); 2016–2017: 98.1% (105/107)]. ST320 presented 98.4% (182/185) of MDR strains, representing 87.1% (182/209) of all MDR strains in CC320 (Table 3).

CC1118 was the most commonly observed CC from 2005–2009 (pre-PCV10) in both age groups, declining after PCV10 introduction. ST1118 (38.1%, 24/63) and ST2878 (22.2%, 14/63) were the most common STs of CC1118. Most of the CC1118 strains (73.0%, 46/63) had a PEN-SXT resistance pattern, with only 1 MDR strain observed from 2011–2015 (Table 3).

CC276 (9.8%, 39/399) and CC733 (7.3%, 29/399) were, respectively, the third and fourth clones most commonly observed among invasive Spn19A strains. CC276 was related to MDR in all periods (71.8%, 28/39), while CC733 had only STX resistance (79.3%, 23/29).

## Discussion

This study shows a relative increase of IPD caused by Spn19A in all age groups after seven years of PCV10 use in Brazil, especially in children <5 years. An increase in antimicrobial resistance of Spn19A was also observed after vaccination. The Spn19A increase was related to the expansion of the international multidrug-resistant clonal complex CC320 in individuals less than 5 years old and more than 50 years old, which are the age groups with the highest incidence of IPD.

The expansion of CC320 among invasive Spn19A strains was mainly related to the expansion of ST320, the predicted founder of CC320 that differs at only a single locus from a large number of other STs included in CC320 [39].

A study conducted in the city of Porto Alegre, Brazil, 3–4 years after PCV10 introduction (2013–2015) among invasive strains from adults ≥50 years identified 10 strains belonging to ST320 among 13 invasive Spn19A strains [28].

In the USA, during the 1990s the main clonal complex identified among Spn19A strains was CC199 and was related only to the PEN intermediate pattern (MIC 0.12–1.0 μg/ml), which gradually decreased after the introduction of PCV7 [23,40,41]. After six years of PCV7 vaccination in the USA (2006), the MDR-CC320 became predominant (70–71%) among the PEN resistant (MIC ≥2.0 μg/ml) Spn19A population; after eight years of PCV7 use (2008), CC320 was predominant among almost half (49.3%) of all Spn19A strains among children <5 years old [23].

In Canada and Spain, after the introduction of PCV7, and in Colombia, where PCV10 was introduced, CC320/ST320 also increased, becoming the most common CC among the MDR or PEN nonsusceptible (MIC ≥0.12 μg/ml) Spn19A populations after vaccination [42,24,25]. Although CC320 spread after PCV7 vaccination in several countries, it decreased after PCV13 introduction [43].

In addition, reports from South Korea, Israel and Asian countries showed an increase in MDR-CC320 in Spn19A before PCV7 introduction, probably due to the excessive use of antimicrobials in these countries [19,20,44]. Therefore, antimicrobial selection pressure may have an important role in CC320 expansion after PCV introduction.

In contrast, in Norway, a country with restricted antibiotics prescriptions, and in Italy, the PCV7 vaccination led to an increase in Spn19A related to CC199, with most strains characterized as PEN susceptible, demonstrating that antibiotic pressure may be important, but not the only factor, for the emergence of Spn19A [45,11].

ST320 is a double-locus variant of ST236, which belongs to the clone Taiwan^19F^-14 (ST236), a widespread international clone first described as related to serotype 19F [46,47]. According to Pillai et al. and Hiesh et al., ST320-Spn19A is derived from a capsular switch event between the vaccine serotype 19F and the non-PCV7 serotype 19A [42,47].

Interestingly, we identified 3 STs of Spn19A in the post-PCV10 period belonging to different CCs that were identified in Brazil and in other countries before PCV introduction expressing diverse serotypes: ST237, ST4913 and ST387—expressing serotypes 19F, 24F and 23F, respectively [38]. These findings suggest a possible capsular switch and a consequent adaptation of the non-PCV10 serotype 19A.

All these observations indicate that serotype 19A and clonal trends among *S*. *pneumoniae* have probably resulted from a combination of several factors, including vaccine and antimicrobial pressure, population immunity, introduction and spread of genetically fit clones, and maybe other factors [22,48,49].

CC1118, not related to MDR, was the most common CC before PCV10 introduction. Other studies also identified CC1118 in the Spn19A population in the northeastern and southern regions of Brazil before and after vaccination [29,30]. In Colombia, ST1118 was also reported to be related to Spn19A especially before vaccination [25]. In Uruguay, only one ST1118-Spn19A strain isolated in 2001 was reported [38]. This occurrence suggests that CC1118-Spn19A might be decreasing after vaccination.

Two other major genetic groups of invasive Spn19A were identified in this study: CC276, related to multidrug resistance, and CC733, related to PEN susceptibility. The emergence of CC276/ST276 related to PEN intermediate resistance (MIC 0.12–1.0 μg/ml) was the cause of the Spn19A increase in children after PCV7 introduction in France [10]. ST276 is genetically related to ST230, which belongs to CC230; CC230 has been identified as the major MDR clone causing IPD by serotype 19A in Europe [50]. Regarding CC733, it is worth mentioning that, according to the MLST database, this clone has been rarely reported in other countries than Brazil, with only one strain in Germany [38].

This study has some limitations, as our data were obtained from a passive laboratory-based surveillance system. In addition to that, during the years 2010–2012, an improvement of laboratory surveillance occurred with the implementation of a case-control study to evaluate PCV10 effectiveness in children in 10 states in Brazil [26], increasing the strains sent to IAL, especially from nonmeningitis cases and individuals aged over 5 years. This led us to analyze the data over a longer period of time after the PCV10 effectiveness study was finished. Despite these limitations, the present study is based on a national surveillance program that includes a large number of invasive Spn19A strains recovered from the same institutions over the whole study period, allowing a robust analysis of the Spn19A population in the country.

In conclusion, we detected a relative increase in invasive Spn19A strains in Brazil seven years after PCV10 introduction. The increase in Spn19A after vaccination was mostly related to the expansion of the multidrug-resistant CC320. The Spn19A-CC320 expansion was probably related to a combination of factors, such as vaccination and antimicrobial pressure. The present data shows the importance of continuous surveillance to understand the mechanisms responsible for CC320 expansion among the invasive Spn19A population in Brazil.

## Supporting information

S1 Table Distribution of antimicrobial nonsusceptibility of invasive Streptococcus pneumoniae serotype 19A among invasive pneumococcus nonsusceptible in the pre-PCV10 period (2005–2009) and in the post-PCV10 periods (2011–2015 and 2016–1017) in Brazil. (DOCX)
