# Supplementary material for: Expansion of the multidrug-resistant clonal complex 320 among invasive Streptococcus pneumoniae serotype 19A after the introduction of a ten-valent pneumococcal conjugate vaccine in Brazil
Source: PLoS One. 2018 Nov 29;13(11):e0208211. doi: 10.1371/journal.pone.0208211 (PMC6264150; doi:10.1371/journal.pone.0208211)
Supplement: S1 Table — (DOCX) [file pone.0208211.s001.docx]

| **Table S1. Distribution of antimicrobial nonsusceptibility of invasive *Streptococcus pneumoniae* serotype 19A among invasive pneumococcus nonsusceptible in the pre-PCV10 period (2005-2009) and in the post-PCV10 periods (2011-2015 and 2016-1017) in Brazil.** | | | | | | |
| --- | --- | --- | --- | --- | --- | --- |
| Antimicrobial non-susceptibility |  | 2005-2009 |  | 2011-2015 |  | 2016-2017 |
|  |  | no. (%) |  | no. (%) |  | no. (%) |
| PEN I+R | Spn19A | 60 (4.7) |  | 268 (22.8) |  | 216 (46.3) |
|  | Spn | 1,277 |  | 1,176 |  | 466 |
| ERY R | Spn19A | 20 (9.2) |  | 208 (30.4) |  | 195 (45.1) |
|  | Spn | 217 |  | 685 |  | 432 |
| STX I+R | Spn19A | 85 (3.8) |  | 273 (16.3) |  | 220 (39.3) |
|  | Spn | 2,235 |  | 1,671 |  | 560 |
| CHL | Spn19A | 1 (3.8) |  | 1 (2.6) |  | 0 (0.0) |
|  | Spn | 26 |  | 39 |  | 18 |
| MDR | Spn19A | 16 (12.1) |  | 190 (48.7) |  | 175 (80.3) |
|  | Spn* | 132 |  | 390 |  | 218 |

Spn19A: total of invasive *Streptococcus pneumoniae* serotype 19A nonsusceptible; Spn: total of invasive *Streptococcus pneumoniae* nonsusceptible; PEN penicillin; ERY: erythromycin; STX: trimethoprim-sulphamethoxazole; I: intermediate; R: resistant; MDR: multidrug-resistant.

*We considered strains with complete result of antimicrobial susceptibility test for penicillin, erythromycin, trimethoprim-sulphamethoxazole, chloramphenicol and vancomycin.
